# Charge as a Selection Criterion for Translocation through the Nuclear Pore Complex

**DOI:** 10.1371/journal.pcbi.1000747

**Published:** 2010-04-22

**Authors:** Lucy J. Colwell, Michael P. Brenner, Katharina Ribbeck

**Affiliations:** 1 School of Engineering and Applied Sciences, Harvard University, Cambridge, Massachusetts, United States of America; 2 FAS Center for Systems Biology, Harvard University, Cambridge, Massachusetts, United States of America; University of Maryland, United States of America

## Abstract

Nuclear pore complexes (NPCs) are highly selective filters that control the exchange of material between nucleus and cytoplasm. The principles that govern selective filtering by NPCs are not fully understood. Previous studies find that cellular proteins capable of fast translocation through NPCs (transport receptors) are characterized by a high proportion of hydrophobic surface regions. Our analysis finds that transport receptors and their complexes are also highly negatively charged. Moreover, NPC components that constitute the permeability barrier are positively charged. We estimate that electrostatic interactions between a transport receptor and the NPC result in an energy gain of several *k*_B_ *T*, which would enable significantly increased translocation rates of transport receptors relative to other cellular proteins. We suggest that negative charge is an essential criterion for selective passage through the NPC.

## Author Summary

All proteins that move between the cytoplasm and the nucleus must pass through nuclear pore complexes, large aqueous channels around 40nm in diameter. In some cases the nuclear envelope is perforated with several thousand nuclear pore complexes, while in other cases they are few and far between. Macromolecular transport through nuclear pores is highly regulated; an elaborate system, involving the binding and unbinding of accessory proteins (transport receptors), allows regulation of which proteins can pass through the pores. The basic principles that govern this selective filtering are not fully understood. Some proteins pass through the pore without binding to transport receptors, while others require the binding of multiple transport receptors for efficient translocation. How does the pore select which proteins can pass through, and which cannot? This paper carries out a biophysical analysis of the properties of proteins that can translocate through the nuclear pore. We find that proteins capable of fast translocation are highly negatively charged, whereas proteins that cannot pass through the pore are positively charged. Moreover, proteins that constitute the interior of the pore channel itself are net positively charged. This suggests that electrostatic interactions between translocating proteins and the pore are an essential part of the selective filtering mechanism.

## Introduction

The defining feature of eukaryotic cells is the separation of nuclear and cytoplasmic compartments by the nuclear envelope. All nuclear proteins, for example polymerases and transcription factors, are made in the cytoplasm and imported into the nucleus. Conversely, RNAs that function in translation are made inside the nucleus and exported to the cytoplasm. The transport of material between the nucleus and cytoplasm occurs through nuclear pore complexes (NPCs), aqueous channels that are embedded in the nuclear envelope. Translocation through NPCs is fully reversible and uncoupled from NTP hydrolysis [1], [2], [3], [4], [5]. Kinetic measurements demonstrate that a single NPC can selectively translocate nearly one thousand molecules per second [6], [7].

Proteins above 30–40 kDa typically only traverse the NPC at appreciable rates with the aid of dedicated nuclear transport receptors (for reviews see [8], [9], [10], [11]). Nuclear transport receptors are soluble proteins that bind to their substrates and translocate together with them through the NPC channel. Importinβ-like transport receptors mediate the vast majority of nuclear transport, and are typically classified as importins or exportins, depending on whether they mediate nuclear import or export. The importinβ superfamily includes at least 21 members in the human proteome *Homo sapiens*, and 14 in the yeast *Saccharomyces cerevisiae* [8].

The NPC consists of approximately 30 proteins (termed nucleoporins) in *S. cerevisiae*, and roughly the same number in vertebrates [12], [13], [14], [15], [16]. Many nucleoporins contain a series of phenylalanine-rich repeats (FG-repeats), which typically occur within the amino acid motifs FxFG or GLFG (here “x” stands for a variable amino acid; [17], and references therein). The FG-repeats are separated by largely unfolded and hydrophilic spacer sequences [17], [18], [19]. Immuno-electron microscopy data reveal that FG-repeat domains occupy the NPC channel as well as the cytoplasmic and nuclear rim of the NPC [19], [20], [21], [22].

Several research groups have established that relatively specific interactions between transport receptors and FG-repeats within nucleoporins are necessary to facilitate selective translocation through the NPC barrier [23], [24], [25], [26], [27], [28], [29], [30], [31], [32], [33], [34], [35], [36], [37]. Transport receptor-FG-repeat interactions are mediated by specific hydrophobic regions on the surface of the transport receptors [6], [24], [25], [26], [28], [29], [32], [33], [38], [39], [40]. Indeed, the crystal structures of importinβ [41], transportin [42], NTF2 [43], [44], and TAP [45] reveal that their surfaces are characterized by a high proportion of hydrophobic regions.

Here, we observe that nuclear transport receptors carry more negative charge than the majority of cellular proteins. The influence of surface charge on the translocation reaction has not been addressed so far. We note that most components of the selectivity barrier within the NPC are characterized by net positive charge [17], [19], [34]. We calculate that electrostatic interactions between a negatively charged transport receptor and positively charged nucleoporins could result in an energy gain of multiple *k*_B_ *T*. This could help compensate for the energy barrier that transport receptors encounter on translocating through the spatially confined NPC channel. We propose that positively charged nucleoporin domains are an important component of the selective filter and promote the specific translocation of negatively charged transport receptors, while imposing a large energy barrier against translocation of positively charged cellular proteins.

## Results

### Translocation competent particles are hydrophobic and negatively charged

**Figure 1:**
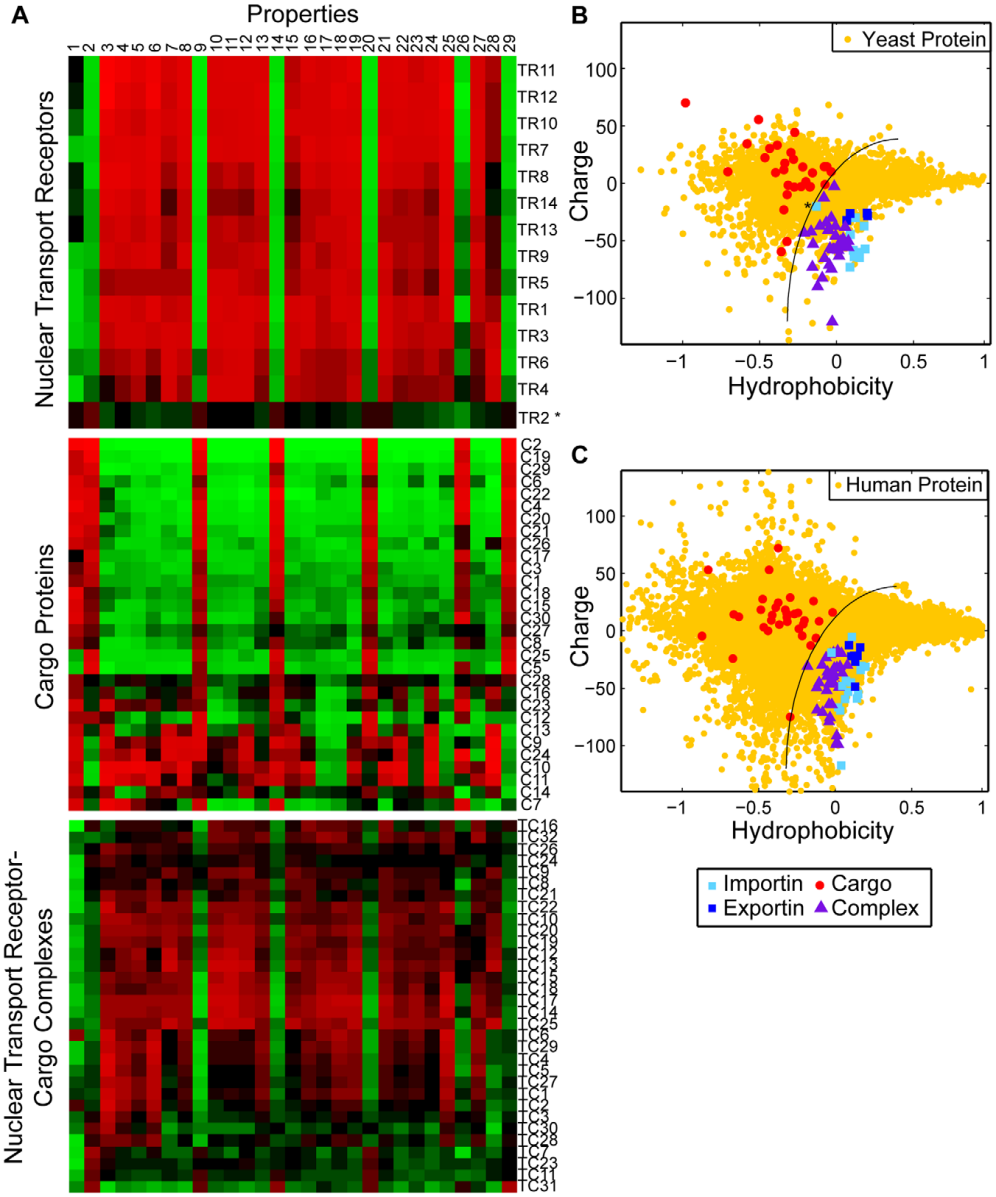
Nuclear transport receptors are more negatively charged than the majority of cellular proteins. A. Heat map of the physical properties of transport receptors, cargo proteins, and transport receptor-cargo complexes from *S.c.* Each row corresponds to a different protein or complex (Table S1), and each column to a different property (Table S2). The value of each property was obtained as described in the main text. Similar proteins are clustered using a Euclidean distance metric. The profiles of transport receptors resemble each other, but differ from those of cargo proteins. B. Reduced representation using the first principal component of the 27 hydrophobicity scales, and net charge at intracellular pH. We compare importins (light blue squares), exportins (dark blue squares), cargo proteins (red circles), and transport receptor-cargo complexes (purple triangles) from *S.c.* with the entire *S.c.* proteome (yellow circles). Transport receptors are characterized by high hydrophobicity and net negative charge, and reside at the edge of the *S.c.* proteome. In contrast, most cargos have comparably low hydrophobicity and net positive charge. Note that the light blue square corresponding to importinα (marked by an asterisk) falls in the region occupied by transport receptor-cargo complexes: indeed, efficient translocation of importinα requires binding to the transport receptors importinβ or CAS, respectively C. Reduced representation of proteins from *H.s.* We compare importins (light blue squares), exportins (dark blue squares), cargo proteins (red circles), and transport receptor-cargo complexes (purple triangles) with the entire *H.s.* proteome (yellow circles).

To understand the biophysical properties that facilitate NPC translocation we first compared the amino acid composition of translocation-competent particles (nuclear transport receptors and cognate transport receptor-cargo complexes) with that of cargo proteins. We assembled a collection of nuclear transport receptors and their cognate cargos from both *S. cerevisiae* and *H. sapiens* (Table S1). In addition, we compiled a list of biophysical properties including measures of charge, polarity, and 27 different empirical metrics for hydrophobicity (Table S2). For every protein, the value of each property (with the exception of the isoelectric point) is obtained by summing the contribution from each amino acid in its sequence, and normalizing by sequence length. Note that these metrics do not take into account the solvent accessibility of each residue; this is not feasible due to the lack of structural information for many cargo proteins. The results of this analysis are displayed as a heat map in Figure 1A. Every property is normalized over the entire set of proteins to have mean zero. Bright red and green correspond to 3 standard deviations above and below this mean, respectively. The proteins are clustered according to the similarity of their properties. Each column corresponds to a different property and each row to a different protein or protein complex. The individual rows in the top panel of Fig. 1A reveal that the profiles of individual nuclear transport receptors resemble each other, but are visibly different to the profiles of individual cargo proteins (middle panel, Fig. 1A). This suggests that particles with translocation-promoting properties are distinct from the average cargo protein with regard to the properties measured here.

To eliminate redundancy in the set of physical properties, we applied principal component analysis (PCA) to the 27 hydrophobicity scales. PCA transforms a number of correlated variables into a smaller number of uncorrelated variables called principal components, ordered according to the amount of variability that they explain.

For the 27 hydrophobicity scales, the first principal component captures 75% of the total variance, and thus serves as an “aggregate” hydrophobicity metric that correlates with each of the individual hydrophobicity scales. Moreover, we find that polarity correlates strongly with the aggregate hydrophobicity scale (correlation coefficient r^2^ = −0.93, Fig. S1), and so can be eliminated as an independent property. In summary, the original 29 dimensional property space could be reduced to just two properties, the aggregate hydrophobicity scale and isoelectric point. Since the NPC is an aqueous channel that is freely permeable to ions, we assume that the pH within the NPC channel is comparable to that in the cytoplasm and so report the net charge at pH 7.2 of each protein or protein complex, instead of its isoelectric point hence forth. The charge 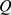 of a protein at pH 7.2 can be predicted from its amino acid sequence according to 
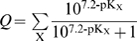
, where the sum is over all ionized amino acids in the protein, and the pK_X_ values were obtained from the *emboss iep* application [46]. We note that representations of protein properties in terms of hydrophobicity and charge have been employed elsewhere [19].

Fig. 1B depicts the hydrophobicity and charge at pH 7.2 of proteins from the yeast proteome. This plot reveals that transport receptors have extreme properties compared to the majority of yeast proteins: Transport receptors and receptor-cargo complexes (light and dark blue squares, and purple triangles, respectively) are collectively more negatively charged at pH 7.2. In contrast, cargo proteins (red circles) tend to be positively charged. The amount of positive charge on a typical cargo protein is far greater than the five to ten charges conferred by a monopartite or bipartite nuclear localization signal [47]. In addition, transport receptors are more hydrophobic than most cellular proteins; as mentioned in the introduction several experiments suggest that specific hydrophobic regions on the surface of nuclear transport receptors are critical for translocation. Note that the segregation of transport receptors from their cargo proteins on the basis of charge and hydrophobicity is also apparent in *H. sapiens* (Fig. 1C and S2). Together, these data suggest that net negative charge at pH 7.2 is an evolutionarily conserved property that distinguishes translocation competent particles from cargo proteins.

### Many nucleoporins that constitute the selectivity barrier are positively charged

**Figure 2:**
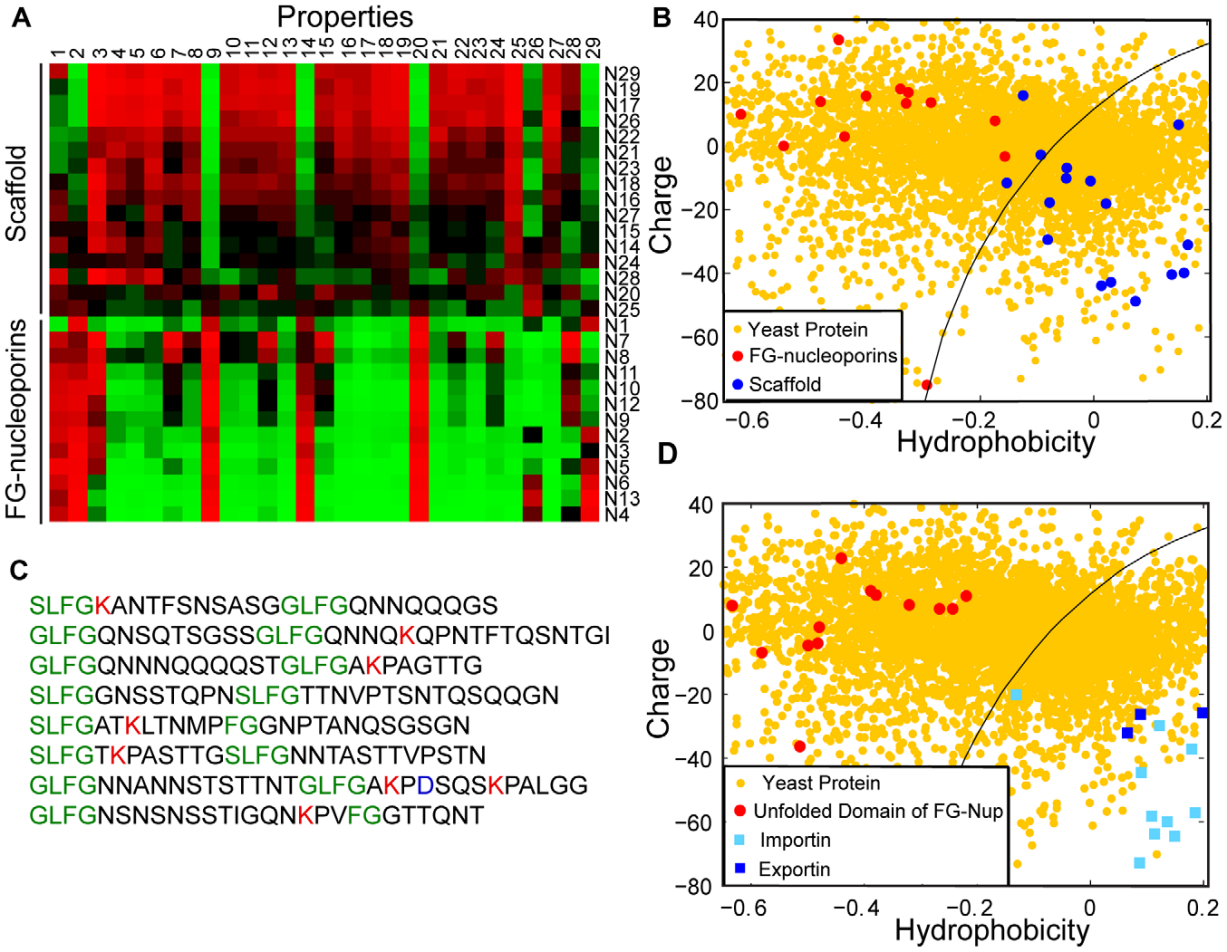
Most components of the NPC selectivity barrier are characterized by net positive charge. A. The physical properties of *S.c.* nucleoporins displayed as a heat map. Each column represents a different property (Table S2), and each row a nucleoporin (Table S3). The value of each property was obtained as described in the main text. Clustering the proteins using a Euclidean metric separates them into two groups, corresponding to scaffold and FG-nucleoporins [17], [22]. B. 2D property space representation of *S.c.* nucleoporins. The hydrophobicity index is plotted against net charge at pH 7.2. The scaffold nucleoporins (blue circles) are relatively hydrophobic, while the FG-nucleoporins (red circles) are characterized by low hydrophobicity and net positive charge. Yellow circles represent the *S.c.* proteome. C. An FG-rich domain from the nucleoporin Nup100. Like other FG-nucleoporins, Nup100 contains largely unstructured FG-domains that contain numerous short hydrophobic FG-repeats separated by hydrophilic, positively-charged linkers. D. Comparison of the unstructured FG-domains [19] (red circles) with transport receptors (light and dark blue squares), and the *S.c.* proteome (yellow circles). The unstructured FG-domains and the transport receptors have complementary properties; the former have low hydrophobicity and mostly net positive charge, the latter have high hydrophobicity and net negative charge at pH 7.2. This suggests a role for electrostatic interactions between transport receptors and the unstructured FG-domains in the translocation reaction.

To address the role of charge in the translocation reaction we next analyzed the amino acid sequences of nucleoporins from *S. cerevisiae* (Fig. 2A; Table S3). Upon clustering according to the similarity of their properties, the nucleoporins segregate into two visually distinct categories, which correspond to their location within the NPC [16], [22]. The top half of Fig. 2A contains structural nucleoporins that form the core scaffold of the NPC. These nucleoporins coat the surface of the nuclear membrane in which the NPC is embedded [16]. The bottom half of Fig. 2A (labeled N1–N13) comprises all nucleoporins that contain FG-repeats. These FG-nucleoporins are anchored to the NPC scaffold and protrude into the inner of the NPC channel, presumably forming the selective barrier [16]. Fig. 2B plots the hydrophobicity and charge at pH 7.2 of the nucleoporins from Fig. 2A. This plot reveals that the majority of FG-nucleoporins (red circles) are characterized by net positive charge and low hydrophobicity, while the scaffold nucleoporins (blue circles) are both more negatively charged and more hydrophobic. Note that mammalian nucleoporins also segregate into distinct groups (Table S3 and Fig. S3).

FG-nucleoporins contain both structured and unstructured domains. The unstructured domains are comprised of numerous hydrophobic FG-repeats separated by hydrophilic spacers [17], [19], [34]. Part of the FG-domain from the yeast nucleoporin Nup100 is shown in Fig. 2C, note that positive charge is present within the hydrophilic spacers that separate the hydrophobic FG-repeats. In this case the positive charge is predominantly derived from the amino acid lysine.

It is presumably the unfolded FG-domains that interact with transport receptors and thus determine selectivity ([25], [28], [29], [36], [37], [39]). In Fig. 2D we analyze the unfolded FG-domains of all 13 FG-nucleoporins (Fig. 2A) as described by [19], [36]. The data suggest that these FG-nucleoporin domains (red circles) are positively charged and hence complementary to the transport receptors (blue squares). This indicates a potential role for electrostatic interactions between the transport receptors and the unfolded domains of the FG-nucleoporins in NPC selectivity.

## Model

### Electrostatic interactions between transport receptors and FG-nucleoporins could result in an energy gain of multiple k_B_T

The rate at which a protein (or protein complex) translocates through the NPC depends upon the energy barrier that it must overcome to enter the NPC [48], [49]. The size of this energy barrier is given by *ΔG = ΔH−TΔS*, where the enthalpy change (*ΔH*) describes the binding energy of a protein to NPC components, *T* is the temperature, and *ΔS* is the change in entropy as a protein enters the NPC. When the charged protein enters the NPC and becomes spatially confined, the entropy of the system decreases, increasing *ΔG* and disfavoring translocation. However, *ΔG* can be lowered if specific interactions between a translocating protein and the NPC decrease enthalpy and thereby compensate for the decrease in entropy.

**Figure 3:**
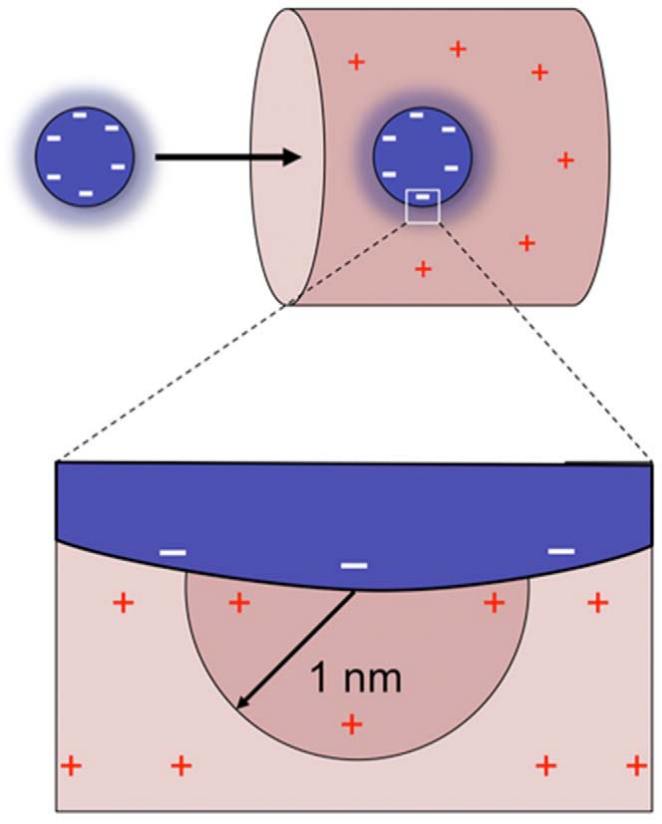
Strategy to estimate the electrostatic interactions between transport receptors and selective barrier components. The interaction between one charge on the surface of the receptor with those nucleoporin charges within a screening length of 1nm (Q_NPC_) is determined. This interaction is then scaled up to include the transport receptor's total charge (Q_NTR_), given by the sum of its charged residues. See main text for details.

We next estimate whether electrostatic interactions between a transport receptor and the NPC interior could in principle be large enough to compensate for the loss of entropy. Within the cell, proteins are surrounded by counter ions that screen their charge; the screening length within the cytoplasm is estimated to be 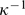
∼1nm [50]. We calculate the size of the interaction between each charge on the surface of the receptor, (where many charged amino acids reside; Fig. S4) and those nucleoporin charges within a hemisphere of radius 1nm (*Q_NPC_*, Fig. 3). We estimate the total charge of the transport receptor (*Q_NTR_*) as the sum of its charged residues.

With this approach the electrostatic interaction energy between a transport receptor and the NPC can be approximated by the Coulombic interaction between *Q_NTR_* and *Q_NPC_*, separated by a screening length 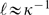:
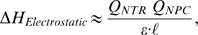
(1)where *Q_NTR_* is the charge derived from the transport receptor, *Q_NPC_* the charge derived from the NPC and 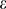 is the dielectric constant of water. A direct derivation of equation (1) follows from assuming the electrostatic interactions are governed by Debye-Huckel theory, and then computing the interaction energy between the transport receptor and a hemisphere of size 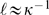 of uniform background charge density filling the nuclear pore. Sophisticated treatments of electrostatic interaction energy are possible [51], [52], and such analyses will likely modify the *ΔH_Electrostatic_* predicted by (1), however they should not change the order of magnitude. For human transport receptors at pH 7.2, we find that 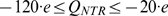, with median 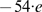, where 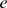 is the electron charge. To calculate *Q_NPC_*, we approximate the yeast NPC as a cylinder with a radius of ∼19nm and a height of ∼37nm [16], and thus a pore volume of 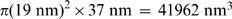. The yeast NPC is thought to contain 13 different types of FG-nucleoporins, with either eight or 16 copies of each [16], [19]. A conservative estimate of the average number of nucleoporins within a hemisphere of radius 1nm is therefore 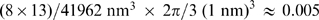. The charge of an individual interior (yeast) nucleoporin ranges from 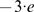 to 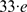, with median 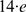, resulting in *Q_NPC_*∼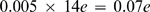.

Equation (1) implies that 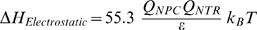, where *Q_NPC_* and *Q_NTR_* are expressed in units of the elementary charge. Using 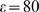 we have that
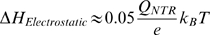
(2)Thus, for a transport receptor with negative charge *Q* = 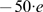, the energy gain due to direct interaction with the NPC interior is *ΔH_Electrostatic_*∼−2.5 *k*_B_ *T*.

We emphasize that the model underlying Equation (2) is simplistic, for example: (i) Our treatment of the electrostatic interactions assumes Debye Huckel interactions, which are quantitatively modified when surface charge densities are sufficiently high; (ii) Detailed structural information for the charge distribution in the NPC channel is not available; (iii) We ignore potential entropic contributions to the electrostatic energy.

Equation (2) thus represents an order of magnitude estimate. Calculation of the entropic cost *ΔS* for a particle to enter the pore is complicated [12], requiring detailed knowledge of the environment both inside and outside the pore [53]. However, it is significant that the *ΔH_Electrostatic_* predicted by Eqn. 2 is the same order of magnitude as *ΔS* [12]. Note that the entropic gain upon leaving the pore will compensate for the ensuing reduction in (electrostatic or other) binding energy [48]. The *ΔH_Electrostatic_* magnitude calculated here would decrease the energy barrier for a transport receptor, increasing translocation efficiency. We therefore propose that electrostatic interactions between negatively charged particles and the positively charged selective barrier components provide a substantial part of the binding energy needed to mediate entry of a particle into the pore.

## Discussion

Our study finds that transport receptors are more negatively charged than the majority of cellular proteins. Existing crystal structures of transport receptors such as importinβ reveal that negative charge is distributed over the surface of the protein (Fig. S4; [40], [45]). High sequence homology of importinβ-like transport receptors, both within species and between species, suggests that net negative surface charge is a conserved property of this protein family.

How could negative surface charge promote the translocation of transport receptors through the NPC channel? Current models of translocation through the NPC postulate that hydrophobic FG-repeats within the selective barrier principally determine NPC selectivity [6], [12], [32], [36], [39], [48], [54], [55]. We suggest that charge within the NPC channel may be a second feature relevant for translocation. The unfolded domains that separate FG-repeats are characterized by net positive charge (Fig. 2C, D), and we suggest that they represent a critical element of the selective barrier. Indeed, the positive charge of the spacers is conserved across multiple species [56], suggesting a functional constraint on the design of the spacer elements.

**Figure 4:**
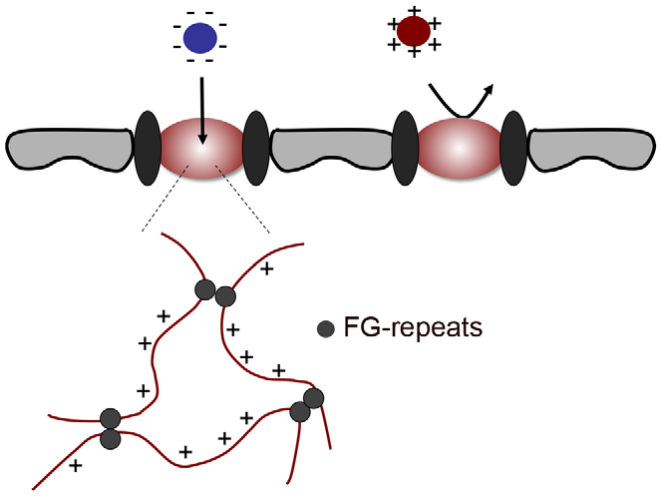
Charge as a selection criterion for nuclear transport. The unfolded domains that constitute the permeability barrier are positively charged (red lines) and generate a positive charge density within the barrier. Particles with negative surface charge (e.g. transport receptors) adsorb to the positively charged nucleoporin domains and thereby selectively partition into the permeability barrier. In contrast, most soluble cellular proteins lack net negative charge; their entry into the NPC is energetically highly unfavorable.

We propose that their negative surface charge allows transport receptors to adsorb to the positively charged nucleoporin domains via electrostatic interactions, facilitating selective partitioning of transport receptors and transport receptor-cargo complexes into the NPC (Fig. 4). Those soluble cellular proteins that are positively charged (Fig. 1B, C) should fail to enter the NPC efficiently because the corresponding energy barrier is too high. Note that according to this model, a translocating particle could become trapped within the NPC if its charge is too negative. The translocation rate is maximal only if the charge of a particle compensates for the decrease in entropy, rendering the total free energy barrier flat. We also note that repulsive electrostatic interactions between the patches of positive charge on the FG-domains may compete with the meshwork forming inter-FG linkages.

Electrostatic interactions as proposed here would help the NPC control entry of particles according to their surface charge, independently of their size, and thereby efficiently hinder passive diffusion of positively charged proteins. This is illustrated by histones, relatively small proteins that do not diffuse efficiently through the pore channel by themselves. Using the simple model in (2) we estimate the contribution of electrostatic interactions to the free energy of translocation for histone1 (H1). H1 has a molecular weight of 21kD and a charge *Q* = 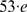 at pH 7.2. Thus it would encounter an energy barrier of ∼2.6 *k*_B_ *T* at the NPC, making spontaneous diffusion highly inefficient. By binding to its two transport receptors, importinβ and importin7 [57], H1 acquires a net charge of 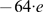, resulting in an energy barrier of ∼−3.2 k_B_T. Combining this electrostatic energy gain with the entropic cost of entering the pore, and possible interactions with FG-repeats, could flatten the energy barrier, thus maximizing the H1 translocation rate.

**Figure 5:**
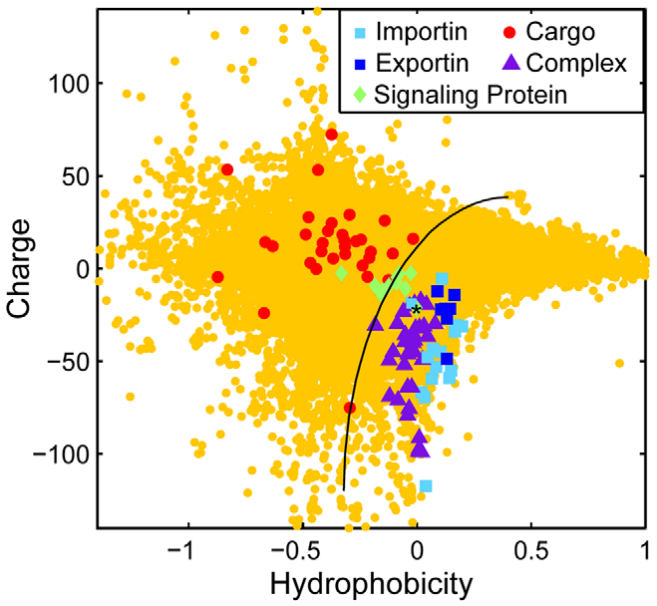
Charge and hydrophobicity of a selection of signaling proteins from *H.s.* (listed in Table S4) (green diamonds) in comparison with importins (light blue squares), exportins (dark blue squares), cargo proteins (red circles), transport receptor-cargo complexes (purple triangles), and the *H.s.* proteome (yellow circles). Signaling proteins that fall into the transport receptor-cargo complex regime (bottom right quadrant) are predicted by this analysis to efficiently self-translocate through the NPC without specific association to transport receptors. Note that beta-catenin (marked with asterisk), one example of a large protein that translocates without binding to a transport receptor, falls in this regime.

Numerous signal transduction molecules are capable of rapidly shuttling between the nucleus and cytoplasm. How their entry into or exit from the nucleus is regulated is a matter of intense investigation. Fig. 5 shows the charge of a small number of well-characterized signaling proteins (listed in Table S4) plotted against their hydrophobicity. Two conclusions can be drawn from this plot. First, a number of shuttling proteins fall in the translocation competent regime, so are predicted to efficiently self-translocate through the NPC without association to transport receptors. One example is beta-catenin (marked with asterisk), which has a molecular weight of 84kDa and is above the passive diffusion limit of the NPC. Indeed, beta-catenin was discovered to translocate through the NPC without associating to a transport receptor [58]. Secondly, a number of signaling proteins, for example STAT3 and SMAD2, fall on the edge of the translocation competence regime. Our analysis predicts that addition of negative charge, as occurs through phosphorylation, could contribute to regulating the translocation rate of such particles. Using Eqn. (2) we estimate that the addition of a single negative charge (corresponding to phosphorylation of a single site) would lead to a decrease in the energy barrier by ∼0.05 *k*_B_ *T*. Thus, phosphorylation of a small number of residues may tune the rate of translocation, even in the absence of a transport receptor.

Specific hydrophobic regions on the surface of nuclear transport receptors are critical for translocation. One important future challenge is to dissect the contribution of both a particle's negative charge and its hydrophobicity to the translocation reaction. This question could be addressed with direct experimental tests that measure a particle's translocation rate as a function of its charge, size and hydrophobicity. The charge and size of particles can be independently varied, and doing so would disentangle their relative contributions to translocation through the pore. The results of such experiments will enable the correlation of a protein's translocation rate through the NPC with its charge and hydrophobicity properties. Moreover, this information could facilitate a novel prediction tool for transport receptor-cargo matching.

## Supporting Information

Figure S1 Correlation between the polarity scale (Grantham) and the aggregate hydrophobicity scale developed in the main text for transport receptors (green squares), complexes (blue triangles), and cargoes (red circles). The correlation coefficient is r^2^ = −0.93.
(0.10 MB TIF)

Figure S2 Heat map of the physical properties of transport receptors, cargo proteins, and transport receptor-cargo complexes in Homo sapiens. Each row corresponds to a different protein or complex (Table S1), and each column to a different property (Table S2). The value of each property (with the exception of the isoelectric point) was obtained for every protein by summing the contribution from every amino acid in its sequence, and normalizing by its sequence length. Each property is normalized to have mean zero over the entire set of proteins. Bright red and green correspond to 3 standard deviations above and below this mean, respectively. Hierarchical clustering, based on a Euclidean distance metric, groups similar proteins together. The profiles of individual transport receptors resemble each other, but are visibly different to the profiles of individual cargo proteins.
(1.27 MB TIF)

Figure S3 A. The physical properties of H.s. nucleoporins displayed in a heat map. Each column represents a different property (Table S2), and each row represents a different nucleoporin (Table S3). The value of each property was obtained as in Fig. 1. Each property is normalized to have mean zero over the entire set of proteins; bright red and green correspond to 3 standard deviations above and below this mean, respectively. Clustering the proteins using a Euclidean metric separates them into two major groups. This plot reveals that FG-nucleoporins are biophysically distinct from other, possibly structural, nucleoporins. Gle1 (N23, marked with an asterisk), does not contain FG-repeats but appears to have similar properties to FG-nucleoporins. B. 2D property space representation of H.s. nucleoporins in context of the human proteome. The hydrophobicity index (the first principal component of the 27 hydrophobicity scales) is plotted against the net charge at intracellular pH. A subset of nucleoporins is characterized by low hydrophobicity and net positive charge, while the other group is relatively hydrophobic. C. 2D property space representation of H.s. nucleoporins. As is the case for the yeast NPC, many human FG-nucleoporins carry net positive charge and are relatively hydrophilic, while most other nucleoporins are collectively net negatively charged and more hydrophobic. The localization of nucleoporins on this 2D plot may allow predictions concerning their localization within the NPC.
(1.15 MB TIF)

Figure S4 Crystal structure of importinβ [40], [45], with negative residues (aspartic and glutamic acid) highlighted in blue. This structure reveals that the negative charge is distributed over the surface of the protein. High sequence homology of importinβ-like transport receptors, both within species and between species, suggests that net negative surface charge is a conserved property of this protein family.
(0.70 MB TIF)

Table S1 Compilation of nuclear transport receptors, cargos, and cognate transport receptorcargo complexes from *Saccharomyces cerevisiae* (A, B) and *Homo sapiens* (C, D) as analyzed in this manuscript.
(0.28 MB DOC)

Table S2 Compilation of biophysical properties analyzed in this manuscript
(0.12 MB DOC)

Table S3 Compilation of yeast and human nucleoporins analyzed in this manuscript.
(0.20 MB DOC)

Table S4 Signaling proteins from *Homo sapiens* analyzed in this paper.
(0.06 MB DOC)
