# Supplementary figures and images for: Charge as a Selection Criterion for Translocation through the Nuclear Pore Complex
Source: PLoS Comput Biol. 2010 Apr 22;6(4):e1000747. doi: 10.1371/journal.pcbi.1000747 (PMC2858669; doi:10.1371/journal.pcbi.1000747)

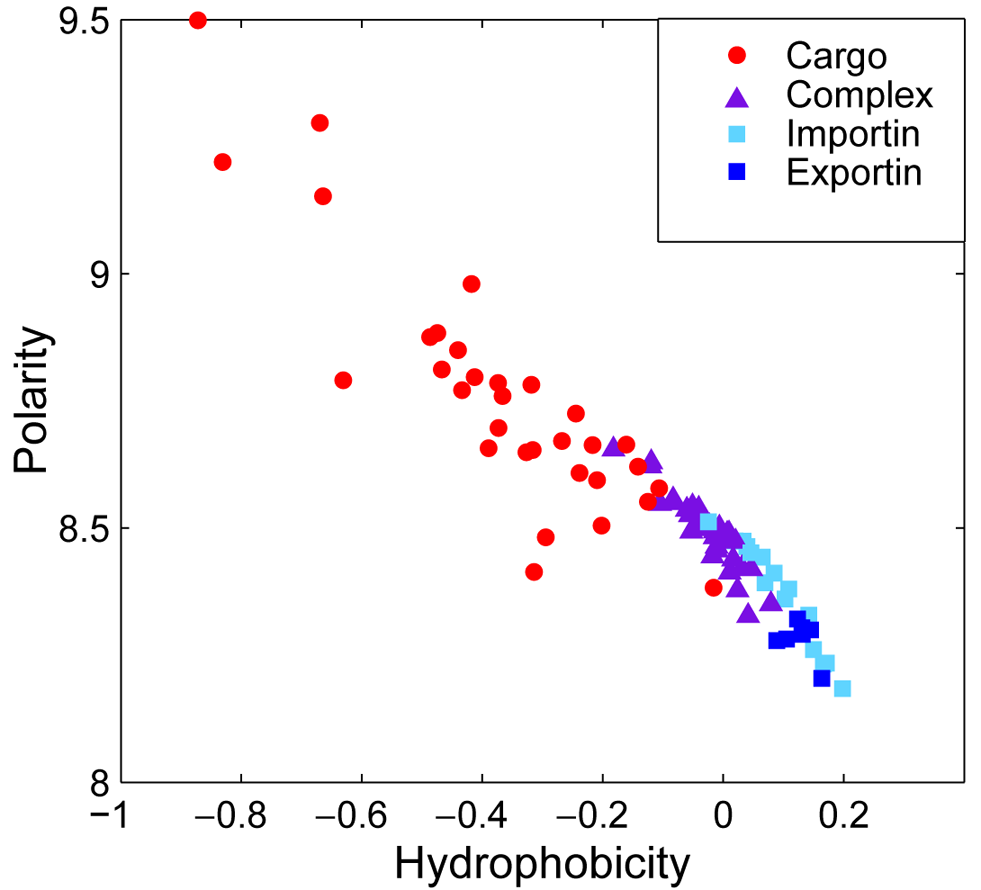

Supplement: Figure S1 — Correlation between the polarity scale (Grantham) and the aggregate hydrophobicity scale developed in the main text for transport receptors (green squares), complexes (blue triangles), and cargoes (red circles). The correlation coefficient is r2 = −0.93. (0.10 MB TIF) [file pcbi.1000747.s001.tif]

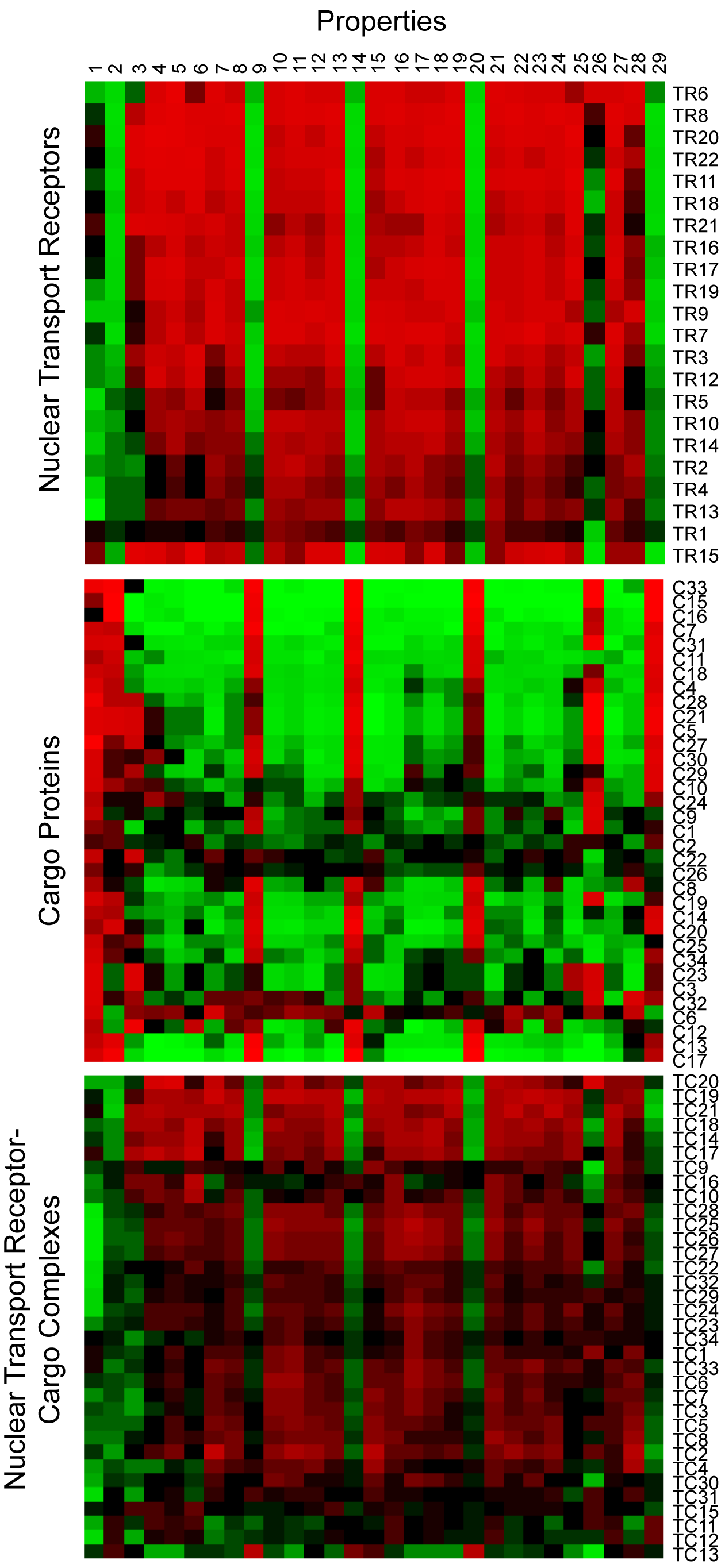

Supplement: Figure S2 — Heat map of the physical properties of transport receptors, cargo proteins, and transport receptor-cargo complexes in Homo sapiens. Each row corresponds to a different protein or complex (Table S1), and each column to a different property (Table S2). The value of each property (with the exception of the isoelectric point) was obtained for every protein by summing the contribution from every amino acid in its sequence, and normalizing by its sequence length. Each property is normalized to have mean zero over the entire set of proteins. Bright red and green correspond to 3 standard deviations above and below this mean, respectively. Hierarchical clustering, based on a Euclidean distance metric, groups similar proteins together. The profiles of individual transport receptors resemble each other, but are visibly different to the profiles of individual cargo proteins. (1.27 MB TIF) [file pcbi.1000747.s002.tif]

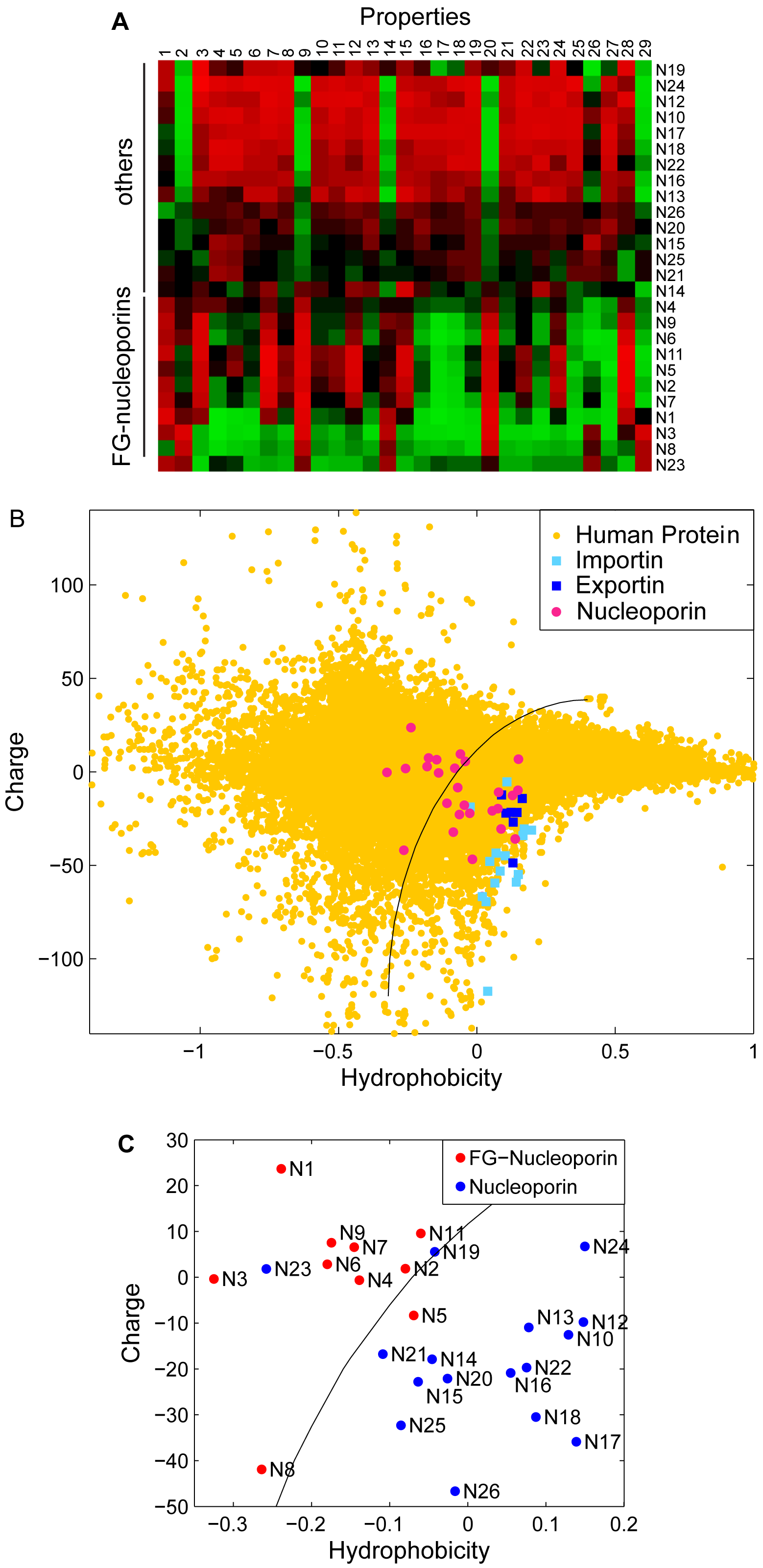

Supplement: Figure S3 — A. The physical properties of H.s. nucleoporins displayed in a heat map. Each column represents a different property (Table S2), and each row represents a different nucleoporin (Table S3). The value of each property was obtained as in Fig. 1. Each property is normalized to have mean zero over the entire set of proteins; bright red and green correspond to 3 standard deviations above and below this mean, respectively. Clustering the proteins using a Euclidean metric separates them into two major groups. This plot reveals that FG-nucleoporins are biophysically distinct from other, possibly structural, nucleoporins. Gle1 (N23, marked with an asterisk), does not contain FG-repeats but appears to have similar properties to FG-nucleoporins. B. 2D property space representation of H.s. nucleoporins in context of the human proteome. The hydrophobicity index (the first principal component of the 27 hydrophobicity scales) is plotted against the net charge at intracellular pH. A subset of nucleoporins is characterized by low hydrophobicity and net positive charge, while the other group is relatively hydrophobic. C. 2D property space representation of H.s. nucleoporins. As is the case for the yeast NPC, many human FG-nucleoporins carry net positive charge and are relatively hydrophilic, while most other nucleoporins are collectively net negatively charged and more hydrophobic. The localization of nucleoporins on this 2D plot may allow predictions concerning their localization within the NPC. (1.15 MB TIF) [file pcbi.1000747.s003.tif]

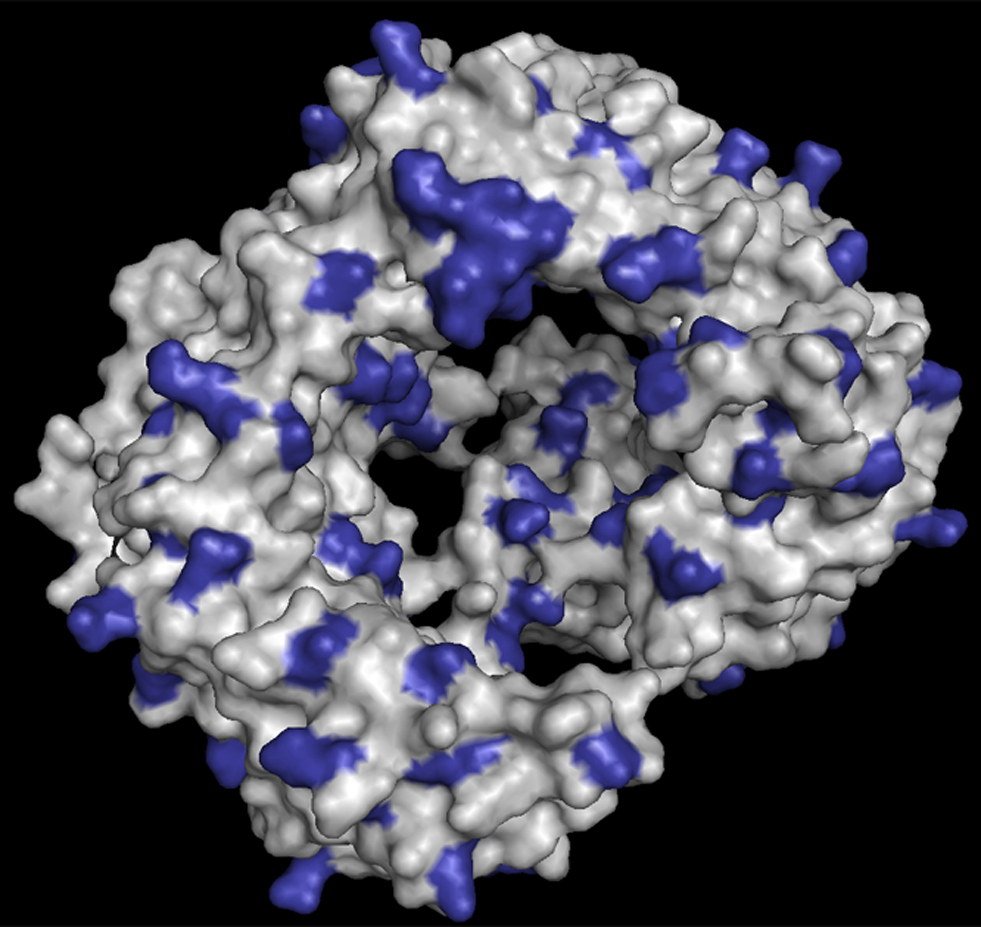

Supplement: Figure S4 — Crystal structure of importinβ [40], [45], with negative residues (aspartic and glutamic acid) highlighted in blue. This structure reveals that the negative charge is distributed over the surface of the protein. High sequence homology of importinβ-like transport receptors, both within species and between species, suggests that net negative surface charge is a conserved property of this protein family. (0.70 MB TIF) [file pcbi.1000747.s004.tif]
