# Supplementary material for: Charge as a Selection Criterion for Translocation through the Nuclear Pore Complex
Source: PLoS Comput Biol. 2010 Apr 22;6(4):e1000747. doi: 10.1371/journal.pcbi.1000747 (PMC2858669; doi:10.1371/journal.pcbi.1000747)
Supplement: Table S1 — Compilation of nuclear transport receptors, cargos, and cognate transport receptorcargo complexes from Saccharomyces cerevisiae (A, B) and Homo sapiens (C, D) as analyzed in this manuscript. (0.28 MB DOC) [file pcbi.1000747.s005.doc]

Tables S1 A-D. Compilation of nuclear transport receptors, cargos, and cognate transport receptor-cargo complexes from *Saccharomyces cerevisiae* (A, B) and *Homo sapiens* (C, D) as analyzed in this manuscript.

A.

| ***Saccharomyces cerevisiae*** | | | | | |
| --- | --- | --- | --- | --- | --- |
| **Nuclear Transport Receptors** | | | **Cargo Proteins** | | |
| **Label** | **Gene** | **Accession** | **Label** | **Gene** | **Accession** |
| TR1 | kap104 | [P38217](http://www.uniprot.org/uniprot/P38217) | C1 | whi5 | Q12416 |
| TR2 | kap60 | [Q02821](http://www.uniprot.org/uniprot/Q02821) | C2 | swi6 | P09959 |
| TR3 | kap121 | [P32337](http://www.uniprot.org/uniprot/P32337) | C3 | far1 | P21268 |
| TR4 | kap123 | [P40069](http://www.uniprot.org/uniprot/P40069) | C4 | crz1 | P53968 |
| TR5 | kap114 | [P53067](http://www.uniprot.org/uniprot/P53067) | C5 | pho4 | P07270 |
| TR6 | kap95 | [Q06142](http://www.uniprot.org/uniprot/Q06142) | C6 | mig1 | P27705 |
| TR7 | kap108 | Q04175 | C7 | aft1 | P22149 |
| TR8 | kap120 | Q02932 | C8 | ste5 | P32917 |
| TR9 | kap119 | P46970 | C9 | maf1 | P41910 |
| TR10 | kap122 | P32767 | C10 | ho | P09932 |
| TR11 | cse1 | P33307 | C11 | hrp1 | Q99383 |
| TR12 | xpot | P33418 | C12 | nab2 | P32505 |
| TR13 | crm1 | P30822 | C13 | prp20 | P21827 |
| TR14 | msn5 | P52918 | C14 | cdc6 | P09119 |
|  |  |  | C15 | swi5 | P08153 |
|  |  |  | C16 | histone 3 | P61830 |
|  |  |  | C17 | histone 4 | P02309 |
|  |  |  | C18 | histone 2a | P04911 |
|  |  |  | C19 | histone 2b | P02293 |
|  |  |  | C20 | nap1 | P25293 |
|  |  |  | C21 | tbp | P13393 |
|  |  |  | C22 | sua7 | P29055 |
|  |  |  | C23 | cdc45 | Q08032 |
|  |  |  | C24 | clb2 | P24869 |
|  |  |  | C25 | spo12 | P17123 |
|  |  |  | C26 | ste12 | P13574 |
|  |  |  | C27 | pdr1 | P12383 |
|  |  |  | C28 | yap1 | P19880 |
|  |  |  | C29 | nop1 | P15646 |
|  |  |  | C30 | sof1 | P33750 |

B.

| ***Saccharomyces cerevisiae*** | | | | |
| --- | --- | --- | --- | --- |
| **Nuclear Transport Receptor-Cargo Complexes** | | | | |
| **Label** | **TR** | **Accession** | **Cargo** | **Accession** |
| TC1 | msn5 | P52918 | pho4 | P07270 |
| TC2 | msn5 | P52918 | mig1 | P27705 |
| TC3 | msn5 | P52918 | aft1 | P22149 |
| TC4 | msn5 | P52918 | ste5 | P32917 |
| TC5 | msn5 | P52918 | maf1 | P41910 |
| TC6 | msn5 | P52918 | ho endonuclease | P09932 |
| TC7 | kap104 | [P38217](http://www.uniprot.org/uniprot/P38217) | hrp1 | Q99383 |
| TC8 | kap104 | [P38217](http://www.uniprot.org/uniprot/P38217) | nab2 | P32505 |
| TC9' | kap95--kap60 | [Q06142--Q02821](http://www.uniprot.org/uniprot/Q06142) | prp20 | P21827 |
| TC10 | kap95--kap60 | [Q06142--Q02821](http://www.uniprot.org/uniprot/Q06142) | cdc6 | P09119 |
| TC11 | kap95--kap60 | [Q06142--Q02821](http://www.uniprot.org/uniprot/Q06142) | swi5 | P08153 |
| TC12 | kap123 | [P40069](http://www.uniprot.org/uniprot/P40069) | histone 3 | P61830 |
| TC13 | kap123 | [P40069](http://www.uniprot.org/uniprot/P40069) | histone 4 | P02309 |
| TC14 | kap114 | [P53067](http://www.uniprot.org/uniprot/P53067) | histone 2a | P04911 |
| TC15 | kap114 | [P53067](http://www.uniprot.org/uniprot/P53067) | histone 2b | P02293 |
| TC16 | kap114 | [P53067](http://www.uniprot.org/uniprot/P53067) | nap1 | P25293 |
| TC17 | kap114 | [P53067](http://www.uniprot.org/uniprot/P53067) | tbp | P13393 |
| TC18 | kap114 | [P53067](http://www.uniprot.org/uniprot/P53067) | sua7 | P29055 |
| TC19 | kap121 | [P32337](http://www.uniprot.org/uniprot/P32337) | pho4 | P07270 |
| TC20 | kap121 | [P32337](http://www.uniprot.org/uniprot/P32337) | spo12 | P17123 |
| TC21 | kap121 | [P32337](http://www.uniprot.org/uniprot/P32337) | ste12 | P13574 |
| TC22 | kap121 | [P32337](http://www.uniprot.org/uniprot/P32337) | pdr1 | P12383 |
| TC23 | kap121 | [P32337](http://www.uniprot.org/uniprot/P32337) | yap1 | P19880 |
| TC24 | kap121 | [P32337](http://www.uniprot.org/uniprot/P32337) | aft1 | P22149 |
| TC25 | kap121 | [P32337](http://www.uniprot.org/uniprot/P32337) | nop1 | P15646 |
| TC26 | kap121 | [P32337](http://www.uniprot.org/uniprot/P32337) | sof1 | P33750 |
| TC27 | msn5 | P52918 | whi5 | Q12416 |
| TC28 | msn5 | P52918 | swi6 | P09959 |
| TC29 | msn5 | P52918 | far1 | P21268 |
| TC30 | msn5 | P52918 | crz1 | P53968 |
| TC31 | kap95--kap60 | [Q06142--Q02821](http://www.uniprot.org/uniprot/Q06142) | cdc45 | Q08032 |
| TC32 | kap95--kap60 | [Q06142--Q02821](http://www.uniprot.org/uniprot/Q06142) | clb2 | P24869 |

C.

| ***Homo sapiens*** | | | | | | | |
| --- | --- | --- | --- | --- | --- | --- | --- |
| **Nuclear Transport Receptors** | | | | **Cargo Proteins** | | | |
| **Label** | **Protein** | **Gene** | **Accession** | **Label** | **Gene** | **Accession** | **Cargo Origin** |
| TR1 | Importin α | kpna1 | P52294 | C1 | hdac4 | P56524 | *H. s.* |
| TR2 | Importin β | kpnb1 | Q14974 | C2 | Large T antigen | [P03070](http://www.uniprot.org/uniprot/P03070) | *Simian virus 40* |
| TR2 | Transportin | tnpo1 | Q92973 | C3 | rpl18a | [Q02543](http://www.uniprot.org/uniprot/Q02543) | *H. s.* |
| TR4 | Importin α--β | kpna1--kpnb1 |  | C4 | rpl5 | [P46777](http://www.uniprot.org/uniprot/P46777) | *H. s.* |
| TR5 | Importin 7 | ipo7 | [O95373](http://www.uniprot.org/uniprot/O95373) | C5 | rpl23a | [P62750](http://www.uniprot.org/uniprot/P62750) | *H. s.* |
| TR6 | Importin-4 | ipo4 | Q8TEX9 | C6 | srebf2 | [Q12772](http://www.uniprot.org/uniprot/Q12772) | *H. s.* |
| TR7 | Transportin-3 | tnpo3 | [Q9Y5L0](http://www.uniprot.org/uniprot/Q9Y5L0) | C7 | tat | [P04610](http://www.uniprot.org/uniprot/P04610) | *HIV type 1* |
| TR8 | Importin-13 | ipo13 | [O94829](http://www.uniprot.org/uniprot/O94829) | C8 | dmrt1 | [Q9Y5R6](http://www.uniprot.org/uniprot/Q9Y5R6) | *H. s.* |
| TR9 | Importin-9 | ipo9 | [Q96P70](http://www.uniprot.org/uniprot/Q96P70) | C9 | snai1 | [O95863](http://www.uniprot.org/uniprot/O95863) | *H. s.* |
| TR10 | RanBP6 | ranbp6 | O60518 | C10 | rps7 | [P62081](http://www.uniprot.org/uniprot/P62081) | *H. s.* |
| TR11 | Importin-11 | ipo11 | [Q9UI26](http://www.uniprot.org/uniprot/Q9UI26) | C11 | rev | [Q77YF8](http://www.uniprot.org/uniprot/Q77YF8) | *HIV type 1* |
| TR12 | Importin-8 | ipo8 | [O15397](http://www.uniprot.org/uniprot/O15397) | C12 | rex | [Q82233](http://www.uniprot.org/uniprot/Q82233) | *Human T-lymphotropic virus 1* |
| TR13 | Importin 7--β | ipo7--kpnb1 |  | C13 | hnrpa1 | [P09651](http://www.uniprot.org/uniprot/P09651) | *H. s.* |
| TR14 | Importin-5 | ipo5 | [O00410](http://www.uniprot.org/uniprot/O00410) | C14 | khdrbs1 | [Q07666](http://www.uniprot.org/uniprot/Q07666) | *H. s.* |
| TR15 | Ntf-2 | ntf2 | [P61970](http://www.uniprot.org/uniprot/P61970) | C15 | pqbp1 | [O60828](http://www.uniprot.org/uniprot/O60828) | *H. s.* |
| TR16 | Exportin-5 | xpo5 | [Q9HAV4](http://www.uniprot.org/uniprot/Q9HAV4) | C16 | hexim1 | [O94992](http://www.uniprot.org/uniprot/O94992) | *H. s.* |
| TR17 | Exportin-t | xpot | [O43592](http://www.uniprot.org/uniprot/O43592) | C17 | fus | [P35637](http://www.uniprot.org/uniprot/P35637) | *H. s.* |
| TR18 | Exportin-1 | xpo1 (crm1) | [O14980](http://www.uniprot.org/uniprot/O14980) | C18 | srp19 | [P09132](http://www.uniprot.org/uniprot/P09132) | *H. s.* |
| TR19 | Exportin-4 | xpo4 | [Q9C0E2](http://www.uniprot.org/uniprot/Q9C0E2) | C19 | ccnt1 | [O60563](http://www.uniprot.org/uniprot/O60563) | *H. s.* |
| TR20 | Exportin-6 | xpo6 | [Q96QU8](http://www.uniprot.org/uniprot/Q96QU8) | C20 | cpsf6 | [Q16630](http://www.uniprot.org/uniprot/Q16630) | *H. s.* |
| TR21 | Exportin-7 | xpo7 | Q9UIA9 | C21 | rpl23a | [P62750](http://www.uniprot.org/uniprot/P62750) | *H. s.* |
| TR22 | Exportin-2 | cse1l (cas) | [P55060](http://www.uniprot.org/uniprot/P55060) | C22 | elavl1 | [Q15717](http://www.uniprot.org/uniprot/Q15717) | *H. s.* |
|  |  |  |  | C23 | rpl18a | [Q02543](http://www.uniprot.org/uniprot/Q02543) | *H. s.* |
|  |  |  |  | C24 | ul84 | [P16727](http://www.uniprot.org/uniprot/P16727) | *Human cytomegalovirus*  *(strain AD169) (HHV-5)*  *(Human herpesvirus 5)* |
|  |  |  |  | C25 | pax6 | [P26367](http://www.uniprot.org/uniprot/P26367) | *H. s.* |
|  |  |  |  | C26 | nr3c1 | [P04150](http://www.uniprot.org/uniprot/P04150) | *H. s.* |
|  |  |  |  | C27 | rpl4 | [P36578](http://www.uniprot.org/uniprot/P36578) | *H. s.* |
|  |  |  |  | C28 | rpl6 | [Q02878](http://www.uniprot.org/uniprot/Q02878) | *H. s.* |
|  |  |  |  | C29 | h4 | [P62805](http://www.uniprot.org/uniprot/P62805) | *H. s.* |
|  |  |  |  | C30 | h3.1 | [P68431](http://www.uniprot.org/uniprot/P68431) | *H. s.* |
|  |  |  |  | C31 | h2b | [P62807](http://www.uniprot.org/uniprot/P62807) | *H. s.* |
|  |  |  |  | C32 | h2a.1 | [O75367](http://www.uniprot.org/uniprot/O75367) | *H. s.* |
|  |  |  |  | C33 | h1 | [P07305](http://www.uniprot.org/uniprot/P07305) | *H. s.* |
|  |  |  |  | C34 | np | [P18277](http://www.uniprot.org/uniprot/P18277) | Influenza A virus  (strain A/Anas; acuta/Primorje/695/1976 H2N3) |

D.

| ***Homo sapiens*** | | | | | |
| --- | --- | --- | --- | --- | --- |
| **Nuclear Transport Receptor-Cargo Complexes** | | | | | |
| **Label** | **Receptor** | **Cargo** | **Gene** | **Accession** | **Cargo Species** |
| TC1 | α--β | Histone deacetylase 4 | hdac4 | P56524 | H. s. |
| TC2 | α--β | Large T antigen |  | [P03070](http://www.uniprot.org/uniprot/P03070) | Simian virus 40 |
| TC3 | Importin β | 60S ribosomal protein L18a | rpl18a | [Q02543](http://www.uniprot.org/uniprot/Q02543) | H. s. |
| TC4 | Importin β | 60S ribosomal protein L5 | rpl5 | [P46777](http://www.uniprot.org/uniprot/P46777) | H. s. |
| TC5 | Importin β | 60S ribosomal protein L23a | rpl23a | [P62750](http://www.uniprot.org/uniprot/P62750) | H. s. |
| TC6 | Importin β | Sterol regulatory element-binding protein 2 | srebf2 | [Q12772](http://www.uniprot.org/uniprot/Q12772) | H. s. |
| TC7 | Importin β | Protein Tat | tat | [P04610](http://www.uniprot.org/uniprot/P04610) | HIV type 1 |
| TC8 | Importin β | Doublesex- and mab-3-related transcription factor 1 | dmrt1 | [Q9Y5R6](http://www.uniprot.org/uniprot/Q9Y5R6) | H. s. |
| TC9 | Importin β | Zinc finger protein SNAI1 | snai1 | [O95863](http://www.uniprot.org/uniprot/O95863) | H. s. |
| TC10 | Importin β | 40S ribosomal protein S7 | rps7 | [P62081](http://www.uniprot.org/uniprot/P62081) | H. s. |
| TC11 | Importin β | Rev | rev | [Q77YF8](http://www.uniprot.org/uniprot/Q77YF8) | HIV1 |
| TC12 | Importin β | Rex | rex | [Q82233](http://www.uniprot.org/uniprot/Q82233) | Human T-lymphotropic virus 1 |
| TC13 | Transportin | Heterogeneous nuclear ribonucleoprotein A1 | hnrpa1 | [P09651](http://www.uniprot.org/uniprot/P09651) | H. s. |
| TC14 | Transportin | KH domain-containing, RNA-binding, signal transduction-associated protein 1 | khdrbs1 | [Q07666](http://www.uniprot.org/uniprot/Q07666) | H. s. |
| TC15 | Transportin | Polyglutamine-binding protein 1 | pqbp1 | [O60828](http://www.uniprot.org/uniprot/O60828) | H. s. |
| TC16 | Transportin | Protein HEXIM1 | hexim1 | [O94992](http://www.uniprot.org/uniprot/O94992) | H. s. |
| TC17 | Transportin | RNA-binding protein FUS | fus | [P35637](http://www.uniprot.org/uniprot/P35637) | H. s. |
| TC18 | Transportin | Signal recognition particle 19 kDa protein | srp19 | [P09132](http://www.uniprot.org/uniprot/P09132) | H. s. |
| TC19 | Transportin | Cyclin-T1 | ccnt1 | [O60563](http://www.uniprot.org/uniprot/O60563) | H. s. |
| TC20 | Transportin | Cleavage and polyadenylation specificity factor subunit 6 | cpsf6 | [Q16630](http://www.uniprot.org/uniprot/Q16630) | H. s. |
| TC21 | Transportin | 60S ribosomal protein L23a | rpl23a | [P62750](http://www.uniprot.org/uniprot/P62750) | H. s. |
| TC22 | Transportin | ELAV-like protein 1 | elavl1 | [Q15717](http://www.uniprot.org/uniprot/Q15717) | H. s. |
| TC23 | Importin-9 | 60S ribosomal protein L18a | rpl18a | [Q02543](http://www.uniprot.org/uniprot/Q02543) | H. s. |
| TC24 | Importin-4 | 65 kDa early nonstructural protein | ul84 | [P16727](http://www.uniprot.org/uniprot/P16727) | Human cytomegalovirus (strain AD169) (HHV-5) (Human herpesvirus 5) |
| TC25 | Importin-13 | Paired box protein Pax-6 | pax6 | [P26367](http://www.uniprot.org/uniprot/P26367) | H. s. |
| TC26 | Importin-7 | Glucocorticoid receptor | nr3c1 | [P04150](http://www.uniprot.org/uniprot/P04150) | H. s. |
| TC27 | Importin  7--β | 60S ribosomal protein L4 | rpl4 | [P36578](http://www.uniprot.org/uniprot/P36578) | H. s. |
| TC28 | Importin  7--β | 60S ribosomal protein L6 | rpl6 | [Q02878](http://www.uniprot.org/uniprot/Q02878) | H. s. |
| TC29 | Importin  7--β | Histone H4 | h4 | [P62805](http://www.uniprot.org/uniprot/P62805) | H. s. |
| TC30 | Importin  7--β | Histone H3.1 | h3.1 | [P68431](http://www.uniprot.org/uniprot/P68431) | H. s. |
| TC31 | Importin  7--β | Histone H2B type 1-C/E/F/G/I | h2b | [P62807](http://www.uniprot.org/uniprot/P62807) | H. s. |
| TC32 | Importin  7--β | Core histone macro-H2A.1 | h2a.1 | [O75367](http://www.uniprot.org/uniprot/O75367) | H. s. |
| TC33 | Importin  7--β | Histone H1.0 | h1 | [P07305](http://www.uniprot.org/uniprot/P07305) | H. s. |
| TC34 | Importin  α--β | Nucleoprotein | np | [P18277](http://www.uniprot.org/uniprot/P18277) | Influenza A virus (strain A/Anas acuta/Primorje/695/1976 H2N3) |

Tables S1 A-D. Compilations of cargo proteins, nuclear transport receptors, and the resulting cognate transport receptor-cargo complexes from *Saccharomyces cerevisiae* and *Homo sapiens*. The values for 29 biophysical properties (Table S2) were obtained for each protein or complex by summing the contribution from each amino acid in its sequence, and normalizing by sequence length. Transport receptor-cargo complexes (Importin α--β, Importin 7--β1) use concatenated sequences of the individual proteins. The results are displayed as heat maps in Figure 1A.
