# Supplementary material for: Charge as a Selection Criterion for Translocation through the Nuclear Pore Complex
Source: PLoS Comput Biol. 2010 Apr 22;6(4):e1000747. doi: 10.1371/journal.pcbi.1000747 (PMC2858669; doi:10.1371/journal.pcbi.1000747)
Supplement: Table S2 — Compilation of biophysical properties analyzed in this manuscript (0.12 MB DOC) [file pcbi.1000747.s006.doc]

Table S2. Compilation of biophysical properties analyzed in this manuscript.

| 1. Polarity 2. Isoelectric Point   *Hydrophobicity Scales:*   1. HPLC retention at pH2.1 [1] 2. HPLC retention at pH 7.4 [1] 3. Retention in HFBA [2] 4. Retention in TFA [2] 5. Rao and Argos [3] 6. Black and Mould [4] 7. Breese et al. [5] 8. Chothia [6] 9. Kyte and Doolittle [7] 10. Eisenberg et al. [8] 11. Fauchere and Pliska [9] 12. Guy [10] 13. Janin [11] 14. Abraham and Leo [12] 15. Manavalan et al. [13] 16. Miyazawa et al. [14] 17. Aboderin [15] 18. Parker et al [16] 19. HPLC at pH3.4 [17] 20. HPLC at pH7.5 [17] 21. Rose et al. [18] 22. Roseman [19] 23. Sweet et al. [20] 24. Welling et al. [21] 25. Wilson et al. [22] 26. Wolfenden et al. [23] 27. Hopp and Woods [24] |
| --- |

Table S1: Biophysical properties used to analyze the yeast and human proteome. We applied principal component analysis to eliminate redundancy in the 27 hydrophobicity scales. The first principal component captures 75% of the total variance for these 27 scales, and thus serves as an “aggregate” hydrophobicity metric that correlates well with each of the individual hydrophobicity scales. In addition, polarity correlates with the aggregate hydrophobicity scale (correlation coefficient r^2^ = - 0.93, see Fig. S1), and can therefore be eliminated as an independent property. In summary, the list of 29 properties could be reduced to the aggregate hydrophobicity scale and isoelectric point. The scales were implemented using *MATLAB’s* Bioinformatic Toolbox.

**References**

1. Meek, J.L., *Prediction of peptide retention times in high-pressure liquid chromatography on the basis of amino acid composition.* Proc Natl Acad Sci U S A, 1980. **77**(3): p. 1632-6.

2. Browne, C.A., H.P. Bennett, and S. Solomon, *The isolation of peptides by high-performance liquid chromatography using predicted elution positions.* Anal Biochem, 1982. **124**(1): p. 201-8.

3. Mohana Rao, J.K. and P. Argos, *A conformational preference parameter to predict helices in integral membrane proteins.* Biochim Biophys Acta, 1986. **869**(2): p. 197-214.

4. Black, S.D. and D.R. Mould, *Development of hydrophobicity parameters to analyze proteins which bear post- or cotranslational modifications.* Anal Biochem, 1991. **193**(1): p. 72-82.

5. Bull, H.B. and K. Breese, *Surface tension of amino acid solutions: a hydrophobicity scale of the amino acid residues.* Arch Biochem Biophys, 1974. **161**(2): p. 665-70.

6. Chothia, C., *The nature of the accessible and buried surfaces in proteins.* J Mol Biol, 1976. **105**(1): p. 1-12.

7. Kyte, J. and R.F. Doolittle, *A simple method for displaying the hydropathic character of a protein.* J Mol Biol, 1982. **157**(1): p. 105-32.

8. Eisenberg, D., et al., *Analysis of membrane and surface protein sequences with the hydrophobic moment plot.* J Mol Biol, 1984. **179**(1): p. 125-42.

9. Fauchere, J.L. and V. Pliska, *Hydrophobic parameters pi of amino acid side chains from the partitioning of N-acetyl-amino acid amides.* Eur J Med Chem, 1983(18): p. 369-375.

10. Guy, H.R., *Amino acid side-chain partition energies and distribution of residues in soluble proteins.* Biophys J, 1985. **47**(1): p. 61-70.

11. Janin, J., *Surface and inside volumes in globular proteins.* Nature, 1979. **277**(5696): p. 491-2.

12. Abraham, D.J. and A.J. Leo, *Extension of the fragment method to calculate amino acid zwitterion and side chain partition coefficients.* Proteins, 1987. **2**(2): p. 130-52.

13. Manavalan, P. and P.K. Ponnuswamy, *Hydrophobic character of amino acid residues in globular proteins.* Nature, 1978. **275**(5681): p. 673-4.

14. Miyazawa, S. and R.L. Jernigan, *Estimation of Effective Interresidue Contact Energies from Protein Crystal Structures: Quasi-Chemical Approximation.* Macromolecules, 1985. **18**: p. 534-552.

15. Aboderin, A.A., *An empirical hydrophobicity scale for a-aminoacids and some of its applications.* Int J Biochem, 1971. **2**: p. 537-544.

16. Parker, J.M., D. Guo, and R.S. Hodges, *New hydrophilicity scale derived from high-performance liquid chromatography peptide retention data: correlation of predicted surface residues with antigenicity and X-ray-derived accessible sites.* Biochemistry, 1986. **25**(19): p. 5425-32.

17. Cowan, R. and R.G. Whittaker, *Hydrophobicity indices for amino acid residues as determined by high-performance liquid chromatography.* Pept Res, 1990. **3**(2): p. 75-80.

18. Rose, G.D., et al., *Hydrophobicity of amino acid residues in globular proteins.* Science, 1985. **229**(4716): p. 834-8.

19. Roseman, M.A., *Hydrophilicity of polar amino acid side-chains is markedly reduced by flanking peptide bonds.* J Mol Biol, 1988. **200**(3): p. 513-22.

20. Sweet, R.M. and D. Eisenberg, *Correlation of sequence hydrophobicities measures similarity in three-dimensional protein structure.* J Mol Biol, 1983. **171**(4): p. 479-88.

21. Welling, G.W., et al., *Prediction of sequential antigenic regions in proteins.* FEBS Lett, 1985. **188**(2): p. 215-8.

22. Wilson, K.J., et al., *The behaviour of peptides on reverse-phase supports during high-pressure liquid chromatography.* Biochem J, 1981. **199**(1): p. 31-41.

23. Wolfenden, R., et al., *Affinities of amino acid side chains for solvent water.* Biochemistry, 1981. **20**(4): p. 849-55.

24. Hopp, T.P. and K.R. Woods, *Prediction of protein antigenic determinants from amino acid sequences.* Proc Natl Acad Sci U S A, 1981. **78**(6): p. 3824-8.
