# Supplementary material for: Charge as a Selection Criterion for Translocation through the Nuclear Pore Complex
Source: PLoS Comput Biol. 2010 Apr 22;6(4):e1000747. doi: 10.1371/journal.pcbi.1000747 (PMC2858669; doi:10.1371/journal.pcbi.1000747)
Supplement: Table S3 — Compilation of yeast and human nucleoporins analyzed in this manuscript. (0.20 MB DOC) [file pcbi.1000747.s007.doc]

Table S3. Compilation of yeast and human nucleoporins analyzed in this manuscript.

| **Nucleoporins (Nups)** | | | | | | |
| --- | --- | --- | --- | --- | --- | --- |
| ***S. cerevisiae*** | | | | ***H. sapiens*** | | |
| **Label** | **Nup** | **Accession** | **Unfolded Domain** | **Label** | **Nup** | **Accession** |
| N1 | 159 | P40477 | 387--1071 | N1 | 153 | P49790 |
| N2 | 53 | Q03790 | 8--253 | N2 | 214 | P35658 |
| N3 | 59 | Q05166 | 1--80 | N3 | 50 | Q9UKX7 |
| N4 | NSP1 | CAA89332 | 1--617 | N4 | 54 | Q7Z3B4 |
| N5 | 1 | P20676 | 121--1076 | N5 | 62 | P37198 |
| N6 | 60 | P39705 | 387--539 | N6 | 98N | P52948 |
| N7 | 42 | P49686 | 1--382 | N7 | Nup like | P52594 |
| N8 | 49 | Q02199 | 1--251 | N8 | 358 | P49792 |
| N9 | 57 | P48837 | 1--255 | N9 | Nup like2 | O15504 |
| N10 | 100 | Q02629 | 1--800 | N10 | 188 | Q5SRE5 |
| N11 | 145N | CAA96798(1:605) | 1--433 | N11 | Nup like1 | Q9BVL2 |
| N12 | 116 | Q02630 | 1--960 | N12 | Pom210 | Q8TEM1 |
| N13 | 2 | P32499 | 52--600 | N13 | 37 | Q8NFH4 |
| N14 | Nic96 | P34077 |  | N14 | 43 | Q8NFH3 |
| N15 | 82 | P40368 |  | N15 | 98C | P52948 |
| N16 | 157 | P40064 |  | N16 | 155 | O75694 |
| N17 | 192 | P47054 |  | N17 | 160 | Q12769 |
| N18 | 170 | P38181 |  | N18 | 205 | Q92621 |
| N19 | 188 | P52593 |  | N19 | 53 | Q8NFH5 |
| N20 | Sec13 | Q04491 |  | N20 | 88 | Q99567 |
| N21 | 84 | P52891 |  | N21 | 93 | Q8N1F7 |
| N22 | 85 | P46673 |  | N22 | 85 | Q9BW27 |
| N23 | 133 | CAA82161 |  | N23 | Gle1 | Q53GS7 |
| N24 | 145C | CAA96798  (606:1317) |  | N24 | Ndc1 | P32500 |
| N25 | Seh1 | P53011 |  | N25 | 107 | P57740 |
| N26 | 120 | P35729 |  | N26 | 133 | Q8WUM0 |
| N27 | Pom152 | P39685 |  |  |  |  |
| N28 | Pom34 | Q12445 |  |  |  |  |
| N29 | Ndc1 | P32500 |  |  |  |  |

Table S3. The values for 29 biophysical properties (Table S2) were determined for each nucleoporin by summing the contribution from each amino acid in its sequence, and normalizing by its sequence length. The results are displayed as heat maps in Figures 2 and S3.
