# Supplementary material for: Charge as a Selection Criterion for Translocation through the Nuclear Pore Complex
Source: PLoS Comput Biol. 2010 Apr 22;6(4):e1000747. doi: 10.1371/journal.pcbi.1000747 (PMC2858669; doi:10.1371/journal.pcbi.1000747)
Supplement: Table S4 — Signaling proteins from Homo sapiens analyzed in this paper. (0.06 MB DOC) [file pcbi.1000747.s008.doc]

**Table S4. Signaling proteins from *Homo sapiens* analyzed in this paper*.***

| **Signaling Proteins**  *(H. sapiens)* | |
| --- | --- |
| **Gene** | **Accession** |
| ctnnb1 | P35222 |
| akt1 | P31749 |
| jnk2 | P45984 |
| p53 | P04637 |
| mapk3 | P27361 |
| stat3 | P40763 |
| stat1 | P42224 |
| stat5 | P42229 |
| smad4 | Q13485 |
| mapk7 | Q13164 |
| smad2 | Q15796 |

Table S4. List of signaling proteins from *Homo sapiens* analyzed in Figure 5. Their charge and hydrophobicity is compared to the human proteome.
